# Zika (PRVABC59) Infection Is Associated with T cell Infiltration and Neurodegeneration in CNS of Immunocompetent Neonatal C57Bl/6 Mice

**DOI:** 10.1371/journal.ppat.1006004

**Published:** 2016-11-17

**Authors:** Mohanraj Manangeeswaran, Derek D. C. Ireland, Daniela Verthelyi

**Affiliations:** Division of Biotechnology Review and Research-III, Office of Biotechnology Products, Center for Drug Evaluation and Research, Food and Drug Administration, Silver Spring, Maryland, United States of America; Washington University, UNITED STATES

## Abstract

The recent spread of Zika virus (ZIKV) and its association with increased rates of Guillain Barre and other neurological disorders as well as congenital defects that include microcephaly has created an urgent need to develop animal models to examine the pathogenesis of the disease and explore the efficacy of potential therapeutics and vaccines. Recently developed infection models for ZIKV utilize mice defective in interferon responses. In this study we establish and characterize a new model of peripheral ZIKV infection using immunocompetent neonatal C57BL/6 mice and compare its clinical progression, virus distribution, immune response, and neuropathology with that of C57BL/6-IFNAR KO mice. We show that while ZIKV infected IFNAR KO mice develop bilateral hind limb paralysis and die 5–6 days post-infection (dpi), immunocompetent B6 WT mice develop signs of neurological disease including unsteady gait, kinetic tremors, severe ataxia and seizures by 13 dpi that subside gradually over 2 weeks. Immunohistochemistry show viral antigen predominantly in cerebellum at the peak of the disease in both models. However, whereas IFNAR KO mice showed infiltration by neutrophils and macrophages and higher expression of *IL-1*, *IL-6* and *Cox2*, B6 WT mice show a cellular infiltration in the CNS composed predominantly of T cells, particularly CD8+ T cells, and increased mRNA expression levels of *IFNg*, *GzmB* and *Prf1* at peak of disease. Lastly, the CNS of B6 WT mice shows evidence of neurodegeneration predominantly in the cerebellum that are less prominent in mice lacking the IFN response possibly due to the difference in cellular infiltrates and rapid progression of the disease in that model. The development of the B6 WT model of ZIKV infection will provide insight into the immunopathology of the virus and facilitate assessments of possible therapeutics and vaccines.

## Author Summary

The recent spread of Zika virus (ZIKV) and its association with increased rates of neurological disorders and congenital defects created an urgent need for animal models to examine the pathogenesis of the disease and explore the efficacy of potential therapeutics and vaccines. We describe the first symptomatic PRVABC59(ZIKV) animal model in immunocompetent B6 WT mice showing that a subcutaneous challenge in 1 day old mice leads to non-lethal neurological disease that is characterized by unsteady gait, kinetic tremors, severe ataxia and seizures that subsides after 2 weeks. ZIKV infects neurons in cerebellum of mice and elicits the infiltration of lymphocytes into the brain. The immune response protects mice from death but may also contribute to neurodegeneration as mice with defective interferon responses have increased virus loads in brain and peripheral organs, succumbing to the disease in 5–6 days, but have fewer signs of neurodegeneration. This mouse model bypasses transplacental transmission and consequent placental insufficiency and will facilitate detailed investigations into the pathogenesis of the disease as well as mechanistic studies for possible therapeutics and vaccines. Lastly, its non-lethal outcome allows for studies assessing the long term effects of the infection, and exploring conditions that could lead to disease reactivation.

## Introduction

Zika virus (ZIKV) is an emerging mosquito-borne pathogen that belongs to the Flavivirus genus of the Flaviviridae family, which includes globally relevant arthropod-transmitted human pathogens such as dengue (DENV), yellow fever (YFV), West Nile (WNV), Japanese encephalitis (JEV), and tick-borne encephalitis viruses. The first strain of ZIKV (MR 766) was isolated in 1947 from a febrile sentinel rhesus monkey in the Zika forest near Entebbe, Uganda after the virus underwent intracerebral passage in Swiss albino mice[1]. For the next 50 years infections with ZIKV were reported sporadically in different regions of Africa and Asia, but were associated with mild symptoms consisting of skin rashes, conjunctivitis, fever and headaches[2]. In 2007 ZIKV started spreading west, first with an outbreak in Island of Yap where it infected over 70% of the population, followed in 2013 by an outbreak in French Polynesia[3]. This last outbreak was associated with a sharp increase in cases of Guillain Barre Syndrome (GBS), an autoimmune disease characterized by weakening and even paralysis of the limbs and face [4,5]. In 2015 Zika spread to South and Central America, infecting thousands of people in Brazil and Colombia, where it associated with an increase in GBS rates as well as a significant increase in severe fetal abnormalities that include spontaneous abortion, stillbirth, hydrocephaly, microcephaly, and placental insufficiency[6–9]. The temporal association of the viral outbreak and increased incidence of GBS and birth defects did not necessarily imply causality, however recent studies showing infection of the CNS in utero as well as infections of human neural progenitor and neural stem cells leading to cell cycle arrest and death [10,11] lend credence to the causality of ZIKV in microcephaly [12,13].

While a majority of ZIKV infections in adults are asymptomatic or result in skin rashes, conjunctivitis (nonpurulent), muscle pain and joint pain (small joints of hands and feet), or a headache lasting for 2–7 days, the recent spread of ZIKV and its association with increased rates of neurological disorders has created an urgent need for animal models to examine the pathogenesis of the disease and explore the efficacy of potential therapeutics and vaccines. In recent weeks, studies showed that young or adult immunocompetent mice are not susceptible to infection, but prepubescent mice lacking the capacity to produce or respond to interferons (IFNs), including A129 (type I IFNAR KO), Interferon Regulatory Factor (IRF)3/5/7 triple KO, and AG129 (type 1 and type 2 IFN KO), develop neurological disease and succumb to infection with high viral loads in the brain, spinal cord, and testes [14,15]. Interestingly, in seeking a model that was less dramatically challenged Lazear et al infected mice deficient in IRF3, IRF5, or Mitochondrial Anti-Viral Signaling (MAVS), which mediate the signaling of pattern recognition receptors for ssRNA RIG-I and MDA5, however none of these mouse models developed disease [14]. While these IFN deficient models provide useful data, the profound immunological defects in these strains may skew our understanding of the pathophysiology of the disease as the impaired IFN response can, for example, modify the susceptibility to infection of specific tissues. Moreover, previous studies on the pathology of flavivirus suggest that pathogenicity may be determined not just by the effect of the virus but by the immune response it elicits [16]. Thus an immunocompetent mouse model is urgently needed to understand the host response and pathogenesis of the disease and to test and compare the potency of potential therapeutic approaches.

More recently several studies have shown that mice from immunocompetent strains can be infected in utero provided a high titer infection is achieved in the dam or antibodies neutralizing interferon are administered [17–20]. These infections, when performed early in pregnancy (E5-E8), result in increased fetal resorption and altered brain and eye development. The relative contribution of virus-induced placental insufficiency versus direct deleterious effect of the virus on the cells of the fetal CNS is unclear [20,21].

In mice, the stage of CNS development of neonatal pups has been equated to a human mid-term fetus [22]. Neonatal rodents have shown to be highly susceptible to many neurotropic viral infections, including Herpes, Bornavirus, Tacaribe arenavirus and more recently Chikungunya, that present with meningoencephalitis [23–26]. In this study we establish a new model of subcutaneous (SC) ZIKV infection in neonatal (1 day old), immunocompetent C57BL/6 (B6 WT) mice and compare its clinical progression, virus distribution, immune response, and neuropathology with C57BL/6-IFNAR^-/-^ (IFNAR KO) mice, which are deficient in type 1 IFN responses. We show that immunocompetent mice, when infected at day 1 of age (P1) develop unsteady gait, loss of balance, kinetic tremors, severe ataxia and seizures beginning around 13 days post infection (dpi) that subside 2 weeks later. Infection-induced IFN responses appear to reduce but not completely abrogate CNS infection in B6 WT mice. Further, whereas the response to the virus in the CNS of B6 WT mice is characterized by cellular infiltration consisting predominantly of CD8+ T cells and is associated with increased expression levels of T cell effector molecules such as IFNg, granzyme B and perforin, the CNS in IFNAR KO mice show infiltration predominantly by neutrophils and macrophages as well as higher levels of inflammatory cytokines. Lastly the CNS of B6 WT mice shows evidence of neurodegeneration that is less prominent in IFNAR KO mice. This model does not address transplacental transmission of virus from mother to fetus or adult transmission, however it offers an immunocompetent symptomatic mouse model for ZIKV infections that may prove useful to understand the long term effects of ZIKV infection. In addition, it avoids transplacental infection and consequent placental insufficiency as a confounding factor in the development of the brain. Lastly, this model may help understand the clinical consequences for infections in late pregnancy or early childhood. While ZIKV infections in early pregnancy have catastrophic consequences, there is concern that infections at later stages of gestation or early childhood may result in long term neurodevelopmental issues that we are as yet unaware of.

## Results

### Susceptibility of Immunocompetent and Immunodeficient Mice to ZIKV

**Fig 1:**
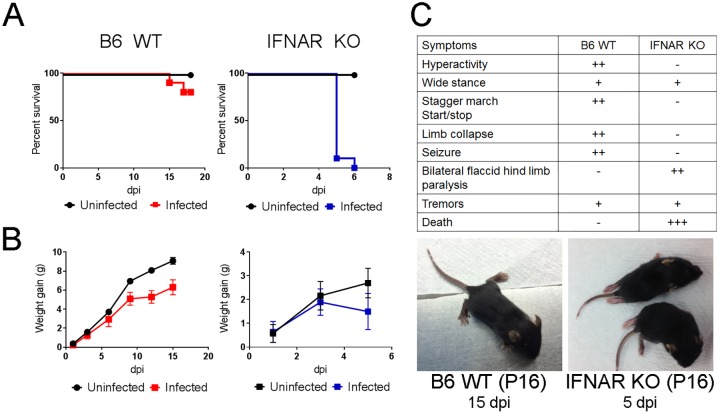
B6 WT mice develop a non-fatal encephalitis following ZIKV infection. **A.** Survival Curves for B6 WT (left, red line, N = 10) and IFNAR KO (right, blue line, N = 10) infected subcutaneously with 2x10^3^TCID50 ZIKV at P1 and P10, respectively. **B.** Weight gain of ZIKV infected B6 WT (N = 7) and IFNAR KO (N = 4) mice. **C**. Clinical presentation in B6 WT and IFNAR KO mice (table) and representative images of 16 day-old (P16) mice at 15 (WT B6) and 5 (IFNAR KO) days post infection (dpi) respectively.

Recent studies showed that mice defective in interferon responses are susceptible to infection and develop a lethal disease. To explore whether IFNAR KO mice are susceptible to infection with the contemporary ZIKV PRVABC59 strain,10 day old (P10) IFNAR KO mice were challenged subcutaneously (sc) with 2 x 10^3^ PFU of ZIKV. The mice remained asymptomatic and maintained their weight gain for the first 4 dpi (Fig 1B). Beginning late on 4 dpi the mice demonstrated reduced movement, tremors, bilateral hind limb paralysis, and died within 24 hours of disease onset (Fig 1). These results were consistent with those reported for 3 week old: A129, IRF-3/5/7 -/- and AG129 (IFNa/b/g KO) mice challenged with the African MR766 or MP1751 strains, or the more recent H/PF/2013 strain [27–29].

We next examined whether the ZIKV PRVABC59 strain could be used to challenge B6 WT mice. Reports on susceptibility to infection *in utero*[30,31], ZIKV’s ability to infect developing neurons *in vitro* [10,17] and prior studies in TCRV[25,32], Sindbis (manuscript in preparation), and Chikungunya [26] suggested that a challenge with ZIKV very early in life, when the central nervous and immune systems are not fully mature may yield a productive infection. Thus we explored whether B6 WT mice were susceptible to ZIKV if challenged one day after birth (P1). As shown in Fig 1, WT mice infected on P1 with PRVABC59 (2x10^3^ PFU (sc)) remain asymptomatic for 12 days, except for a decreased rate of weight gain. After 2 weeks the mice develop unsteady gait with widening stance, hyperactivity and ataxia. This is followed by reduced mobility, intermittent alternating collapse of the hind limbs, loss of balance and seizures (S1–S3 Movies). Interestingly, unlike IFNAR KO mice, these mice do not develop flaccid hind limb paralysis or succumb to the infection (Fig 1 and S4 Movie). Indeed, the observed symptoms diminish over the course of 2 weeks and most mice survive the challenge and recover. Additional studies will be needed to assess the long term consequences of infection.

To determine whether the difference in clinical presentation was due to the age of the mice at the time of challenge, IFNAR KO mice were challenged at P1 and P3. The clinical presentation in mice challenged on P1 or P3 was similar to that of mice challenged on P10 as they developed bilateral paralysis and succumbed to disease by 5 dpi (S1 Fig). Conversely, B6 wt mice challenged on P3 or P10 do not develop signs of disease. This indicated that the virus was inducing fundamentally different pathology in immunocompromised and immunocompetent mice. Given that the clinical development of the disease was similar for mice challenged on P1, P3 or P10, all the ensuing experiments used P10 infections for the IFNAR KO model so that the peak of disease coincides in age with that of the B6 model (P15).

### Tissue distribution of ZIKV in Mice

**Fig 2:**
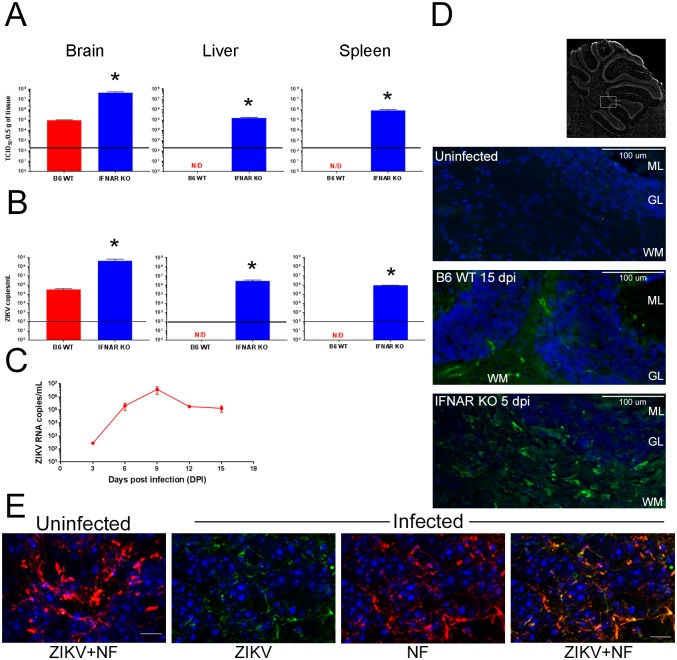
ZIKV infects the CNS of B6 WT animals in the absence of a detectable peripheral infection. **A.** Quantification of infectious virus in the CNS and peripheral organs of B6 WT and IFNAR KO mice. Data is presented as TCID_50_/0.5 g of tissue for each organ (N = 4 per group). N/D = not detectable. **B.** ZIKV RNA was measured in the CNS and peripheral organs of infected B6 WT and IFNAR KO mice (N = 4 per group) using quantitative real-time PCR. Values are presented as the number of viral RNA copies/ml. N/D = not detectable. For A. and B. the horizontal line in each graph denotes the limit of detection for the assay. Statistical differences between groups were assessed using the Mann-Whitey test (* p<0.03). **C**. Depicts the viral RNA levels in CNS of B6 WT mice at 3,6, 9,12 and 15 dpi. **D.** Virus infected cells in the cerebellum of B6 WT and IFNAR KO mice. Epifluorescent images demonstrate ZIKV infected cells (green) in the CNS of both B6 WT (15 dpi) and IFNAR KO mice (5 dpi). As expected, uninfected B6 WT mice (top) did not stain positively for the presence of the virus. Scale bar = 100 μm. **E.** Confocal imaging of neurons in cerebellum of ZIKV infected B6 WT mice stained with antibodies to neurofilament-heavy chain (NF, red) and human polyclonal anti- ZIKV ab (green). Scale bar = 20 μm.

Previous reports show that in mice defective in IFN responses (A129, AG129, IRF-3/5/7 triple KO) the virus distributes systemically, with detectable ZIKV present in the brain and spinal cord, testes, spleen, liver, kidney and serum [28]. Our challenge model with PRVABC59 in IFNAR KO mice confirms these results showing high virus titers in the CNS (4.4 x 10^7^ TCID_50_), as well as spleen (9 x 10^5^ TCID_50_/0.5 g of tissue), and liver (1.5 x 10^5^ TCID_50_/0.5 g of tissue) at 5 dpi (Fig 2A). In comparison, WT mice challenged at P1 show relatively lower viral loads in the CNS (9x10^4^ TCID_50_/0.5 g of tissue) at 15 dpi, the time when the animals displayed peak neurological deficit. Moreover, the B6 WT mice do not show evidence of viral infection in spleen or liver, indicating selective infection of the CNS. Similarly, quantitative real-time PCR showed detectable levels of viral RNA in the CNS of B6 WT mice starting at day 3 and increasing through day 9 of infection (Fig 2B and 2C). Of note the levels of viral RNA in CNS did not reach those evident in IFNAR KO mice at peak of disease (10^5^ ZIKV RNA copies/mL vs 10^8^ ZIKV copies/mL) (Fig 2A and 2B). No RNA was detectable in liver or spleen of B6 mice (Fig 2B). The presence of more than 10^5^ ZIKV RNA copies /mL in liver and spleen of IFNAR KO mice suggests that type I IFNs play a key role in controlling the virus in the peripheral organs and peripheral infection may play a role in lethality in these models. In contrast, infection in B6 WT appears to be restricted to the CNS possibly due to lower levels of IFNs in response to the virus in CNS as previously shown [33,34].

Given that both strains were positive for ZIKV in the CNS but displayed different clinical presentation, we next explored whether the distribution of the virus in the CNS was similar in B6 WT and IFNAR KO mice. Immunohistochemistry of the CNS using a mouse monoclonal antibody (clone D1-4G2-4-15) that has been shown to react with ZIKV and other members of the flavivirus family, demonstrated detectable viral antigen in the CNS of both B6 WT (15 dpi) and IFNAR KO (5 dpi) mice (Fig 2D). As expected, age-matched, uninfected control sections were negative for the virus stain (Fig 2D). Consistent with the higher virus titers, IFNAR KO mice showed stronger and broader staining of the virus by immunohistochemistry that extended to the cortex. In contrast, the CNS of B6WT mice showed virus predominantly in the cerebellar white matter and granular layers as well as in the hippocampus region (S2 Fig), but not in the frontal cortex. Colocalization of stains for ZIKV and neurofilament heavy chain in the areas of cerebellum (Fig 2E) and hippocampus (S2 Fig) indicates that ZIKV infects neurons.

### Characterization of the response to ZIKV infection in the CNS of WT and IFNAR KO mice

**Fig 3:**
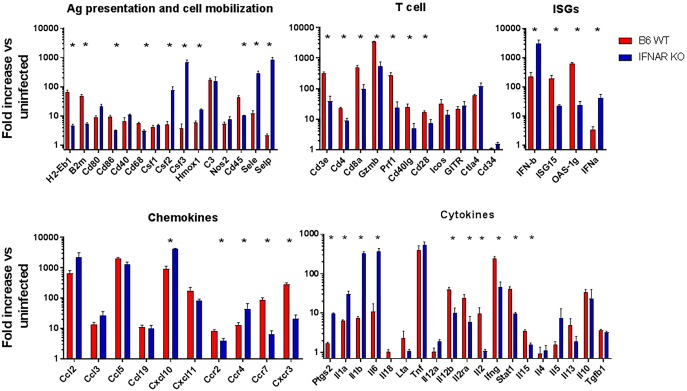
Inflammatory gene expression in the CNS in response to ZIKV infection. Mouse inflammation TLDA analysis comparing ZIKV infected B6 WT (red) and IFNAR KO (blue) CNS at P16 (15 dpi and 5 dpi, respectively). The genes are organized by functional class: Ag presentation, T cell, chemokines and cytokines. Interferon stimulated genes (ISGs) expression was measured using individual Taqman assays performed in triplicate. Data presented as fold increase over age-matched uninfected controls for each strain. N = 4 mice per group representative of two independent experiments, * = p < 0.05.

We next determined whether the immune response to the virus in the CNS was similar between IFNAR KO and B6 WT mice. Both strains showed profound up-regulation of the expression of genes linked to inflammation and cellular infiltration. These included a significant up-regulation of *Ccl2*, *Ccl5*, *Cxcl10* and *Cxcl11* with the corresponding increases in genes linked to the recruitment and activation of neutrophils (PMN) and monocyte/macrophages including increases in MHC, *Cd80*, *Cd86*, *Cd68* and *Cd40* as well as marked increases in Csf1 and Csf2 (Fig 3). These were accompanied by significant increases in the expression of *IFNb*, *Tnfa*, *Il6*, *Il1*, *Ifng*, *C3* and *Cox2*, all indicating a severe inflammatory response in the CNS. In all, 49 of the 96 genes screened showed at least a 5 fold increase in expression in both strains. Interestingly, the magnitude of the up-regulation for several of these genes was markedly different between the models. For example, IFNAR KO mice showed significantly higher levels of *Csf2*, *Csf3*, Sele and Selp, while B6 WT mice showed relatively higher levels of genes linked to antigen presentation such as H2-Eb1 and B2m. B6 WT mice also showed significantly higher levels of *Cd45*, *Ccr7* and *Cxcr3*, suggesting increased infiltration of peripheral leukocytes, while IFNAR KO mice showed higher levels of *Ccr4* likely expressed on microglia and astrocytes (Fig 3). Among the genes linked to inflammation, the expression of *Ifna*, *Ifnb*, *Cox2*, *Il1*, and *Il6* were significantly higher in the IFNAR KO mice potentially due to higher virus titers in the CNS and the deficient IFN response. In contrast, the CNS of B6 WT mice had relatively higher expression of ISGs OAS1 and ISG15, as well as genes corresponding to T cells including CD3, CD4 and CD8, and markers of Th1 and cytolytic responses such as G*zmB* and *Prf1*, *Il2*, *Ifng*, and *STAT1*. These data would indicate that ZIKV enables a significant T cell response in the CNS of B6 WT mice that is not evident in IFNAR KO mice.

### Cellular infiltration in the CNS of ZIKV infected B6 and IFNAR KO mice

**Fig 4:**
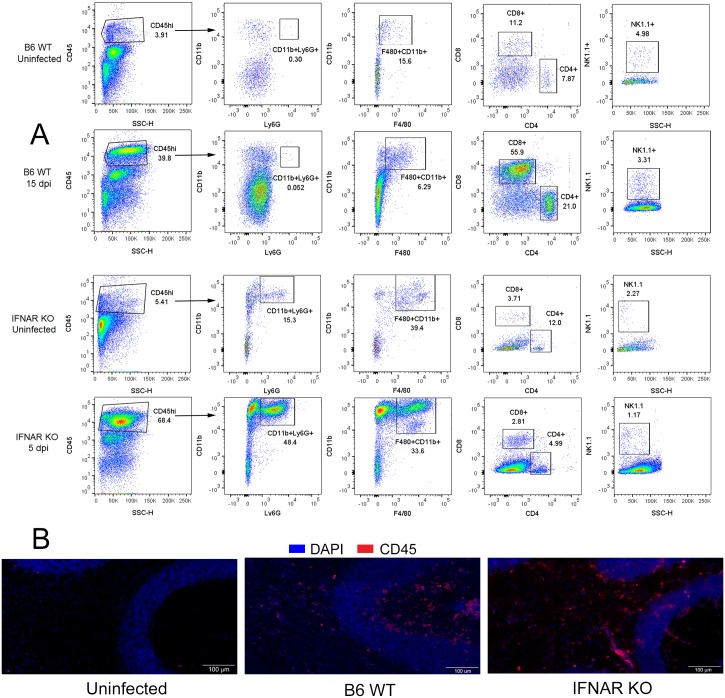
Immune cells infiltrating the CNS in response to ZIKV infection. **A**. Flow cytometry performed on cells isolated from the CNS (N ≥ 5 mice pooled) of ZIKV infected B6 WT and IFNAR KO mice at 15 and 5 dpi, respectively. Live cells were gated and separated based on CD45 expression. Infiltrating CD45hi cells were gated (left column) and the populations of Ly-6G+ neutrophils, CD11b+F4/80+ macrophages, CD4+ and CD8+ T cells and NK1.1+ NK cells, respectively within the CD45hi population were determined. These data are representative of two independent experiments. **B**. IF-IHC staining for CD45 (red) showing that CD45+ immune cells are infiltrating all layers of the cerebellum in both B6 WT and IFNAR KO mice. Uninfected mice showed no staining for CD45^+^ cells. Scale bar = 100 μm.

The data above suggested that the CNS infection in both strains was accompanied by microglial/macrophage activation and immune cell infiltration. To explore this further, we isolated cells from the CNS of infected animals at 15 dpi and studied the cell populations using flow cytometry. As predicted by the increased expression of genes related to T cells and antigen presenting cells, there was significant cellular infiltration of CD45^hi^ cells in the CNS of both strains. In B6 WT mice the majority of infiltrating cells were T cells, with CD8^+^ T cells comprising 45% and CD4+ T cells comprising 20% of the CD45^hi^ infiltrating population. The remaining cell types consisted of F4/80+CD11b+ macrophages (15%), NK1.1+ Natural Killer cells (3%) and CD19+CD45R+ B cells (5%)(Fig 4A). IFNAR KO mice showed even higher levels of cellular infiltration, however in these mice the infiltrating cells corresponded to CD11b+Ly6G+ and CD11b+F4/80+ consistent with PMN and macrophages respectively, with only a minor population of T cells and NK cells (Fig 4A). These data together with the analysis of gene expression suggests that the inflammatory and immune processes that follow ZIKV are fundamentally different in IFNAR KO and B6 WT mice, with IFNAR KO mice showing significant inflammation of the CNS accompanied by infiltration by PMN and granulocytes, while IFN-sufficient animals mount a T cell driven response characterized by CD8+ T cells and high levels of IFNγ and granzyme B.

Previous studies had shown inflammatory and degenerative changes in the brains of IFN deficient mice challenged with ZIKV. These include the presence of scattered nuclear fragments, perivascular cuffing, and PMN infiltrating gray and white matter [27]. To determine whether the B6 WT mice would show similar evidence of inflammatory and degenerative changes, we stained sagittal brain sections collected from Zika-infected B6 WT mice at 15 dpi and age-matched control animals. Fluorescence immunohistochemistry confirmed the presence of CD45+ immune cells in the parenchyma of the CNS in both B6 WT and IFNAR mice and showed that these cells concentrate in the white matter and granular layers of the cerebellum (Fig 4B), consistent with previous studies in IFN deficient mice [27].

### ZIKV infection results in neurodegeneration in the CNS of B6 WT mice

**Fig 5:**
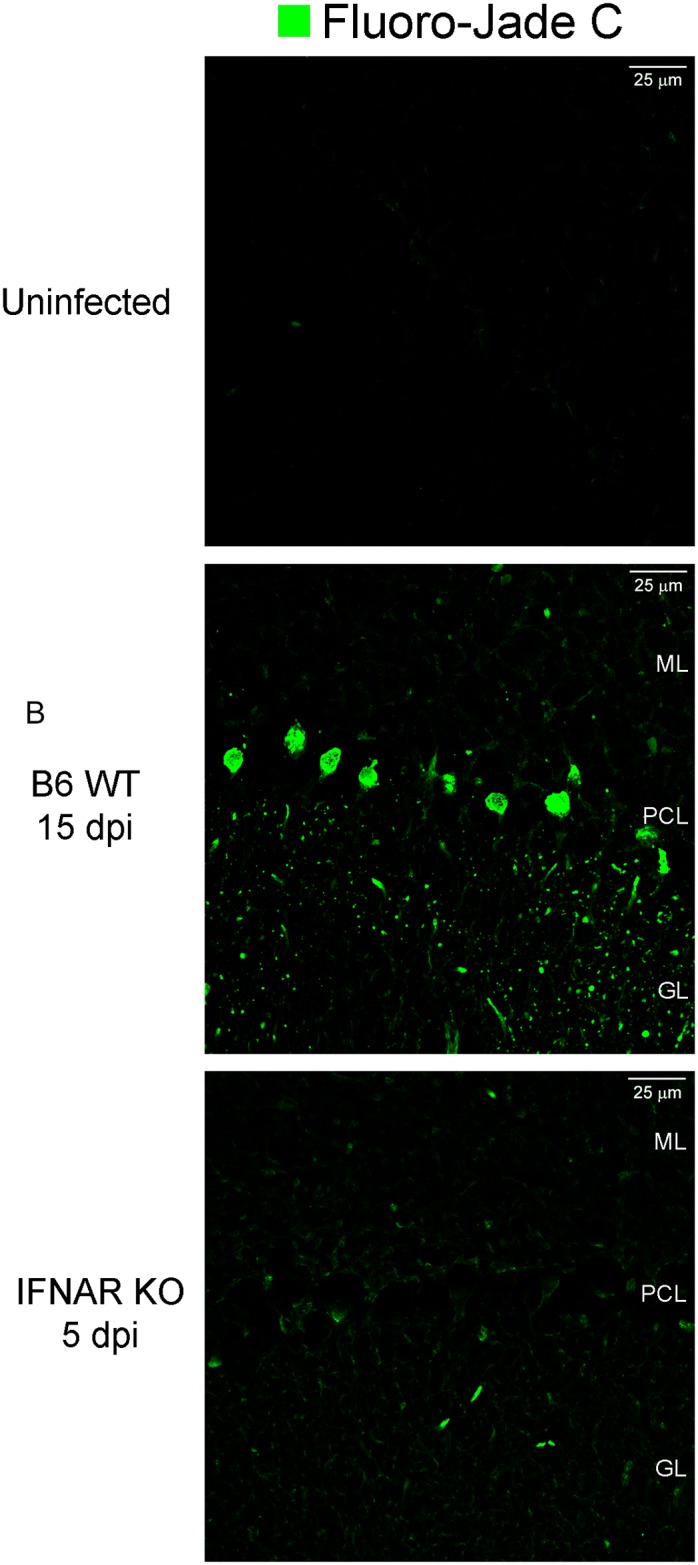
ZIKV infection results in neurodegeneration. Confocal imaging of Fluoro-Jade C histochemical stain (green) shows neurodegeneration in the cerebellum of ZIKV infected mice when compared to the uninfected B6WT CNS (top). Note that the staining with Fluoro-Jade C is minimal in similar regions of the cerebellum of IFNAR KO (5 dpi) mice. Scale bar = 25 μm. GL = granular layer, PCL = Purkinje cell layer, ML = matrix layer.

The clinical presentation of ZIKV infected B6 WT mice, along with the previously described tropism of ZIKV for neurons suggested that the virus infects and damages the CNS of B6 WT mice. To test this, we stained sections adjacent to those used to detect virus and inflammation with the Fluoro-Jade C, a stain that specifically labels degenerating neurons. As expected, uninfected, age-matched controls showed no staining with the Fluoro-Jade C stain. In contrast, infected B6 WT mice showed foci of Fluoro-Jade C positive neurons in all layers of the cerebellum, but predominantly in the granular and PC layers (Fig 5). Interestingly, there were fewer Fluoro-Jade C+ cells in granular and Purkinje layers of the cerebellum of IFNAR KO mice. The presence of infiltrating CD8+ T cells and higher levels of fluorojade C positive cells in the CNS of B6 WT mice suggests a possible role for CD8+ T cells in the pathology.

## Discussion

ZIKV belongs to the Flavivirus genus that includes several etiological agents of viral encephalitis, the most significant being Japanese encephalitis virus, West Nile virus, and tick-borne encephalitis virus. As with other flaviviruses, the majority of infected individuals will not develop disease, but a minority will develop a severe illness with a significant chance of permanent neurological damage, congenital malformations, or death. The factors that determine this are likely numerous, involving complex interactions between virus and host that are yet to be uncovered. Animal models can help us understand the pathophysiology of the virus, identify therapeutic targets, and explore the safety and efficacy of new therapeutics and vaccines. This study shows that neonatal B6 WT mice challenged with ZIKV develop a slow onset non-lethal encephalitis that is characterized by unsteady gait, kinetic tremors, severe ataxia, loss of balance and seizures. The virus localizes to the CNS where it elicits a strong IFN response, T cell infiltration with increased expression of RNA coding for Ifng, granzymeB, perforin1 and Il-2. In addition, these mice show evidence of neurodegeneration in particular affecting the Purkinje and granular cell layers of the cerebellum as evidenced by Fluoro-Jade C staining. Our data suggests that innate and adaptive responses can limit viral expansion but may also play a role in pathological changes in the CNS.

In response to the outbreak of ZIKV and its association with increased congenital and neurological disease, animal models are being developed with unprecedented celerity to understand the pathogenesis of the disease and test possible therapeutics and vaccines. Early studies in mice had suggested that ZIKV can replicate and cause injury in cells of the central nervous system [1,35,36] but used the prototype MR 766 strain of ZIKV, which had undergone extensive passage in suckling mouse brains. Several new mouse models of ZIKV were developed over the past 6 months using current viral isolates. Using a low passage Cambodian isolate of ZIKV, Rossi *et al* showed that 3 week old A129 and AG129 mice, which lack type I or I & II IFN respectively, develop paralysis and succumb to disease by 7 dpi while older mice showed viremia and weight loss but recovered after day 8[15,29]. Similar results were observed by Lazear et al, using either 4–6 week old A129 or Irf3/5/7 triple knockout mice challenged with ZIKV (H/PF/2013) from French Polynesia, as well as African ZIKV strain MR 766[28]. Our studies advance on these findings by characterizing the immune response to ZIKV in the CNS and key peripheral organs. We show that in the CNS of mice with deficient IFN responses the virus in the brain was localized predominantly to the cerebellum at the peak of disease and elicits a marked inflammatory response characterized by significant increases in the mRNA expression of complement (C3), Cox2, Il1a, Il1b, and Il6. The increased mRNA expression of Sele, Selp, Csf2 and Csf3 is consistent with the observed increase in infiltrating neutrophils and macrophages evident by flow cytometry. Together this pattern of expression suggests the activation of microglia and/or infiltration of activated macrophages, which has been shown to lead to uncontrolled inflammation and neuronal death in other models of flavivirus encephalitis such as mice infected with Japanese encephalitis virus [37]. Interestingly, despite significant upregulation of markers for inflammation and the evidence of infiltrating neutrophils and macrophages, the infected IFNAR KO mice did not show significant increase in the expression of genes linked to apoptosis (including BCL2, Bax, Agtr2 or Bcl2l1) (S3 Fig). This differs from studies showing a role for caspase 3 in the apoptosis of infected neurons in vitro [38]. Similarly we found no evidence of widespread neurodegeneration in the CNS, although this could be due to the rapid progression of the disease, or result from the absence of IFN responses, which could sensitize the tissue to apoptosis and support CD8+ T cell mediated cytotoxic responses [39]. Alternatively, recent studies suggest that ZIKV infection results in the formation of autophagosomes that facilitate virus replication [40]. Since autophagy is negatively correlated with Type I interferon production [41], it is possible that the increase in viral load and lack of infiltrating T cells in IFNAR KO mice may be secondary to increased autophagy, reduced antigen clearance by programmed necrosis, and/or reduced presentation. Additional studies will be needed to assess the effects of infiltrating neutrophils and macrophages in the CNS of infected IFNAR KO mice and determine whether they would constitute a therapeutic target in the human disease. Lastly, given the limited neurodegeneration observed in the CNS of IFNAR KO mice at peak of disease and the rapidity of death in the IFNAR KO mice, it is possible that infection of the peripheral organs contribute to the lethality of ZIKV infection in IFNAR KO mice, and thus although the mice show clear signs of neurological damage, additional studies will be needed to establish the cause of death in the infected IFNAR KO mice.

The neonatal B6 WT model presents several striking differences with the IFNAR KO model used in these studies. In addition to the differences in the pace (prodrome of 13 vs 5 days) and survival, the B6 WT model shows no evidence of virus spread into spleen or liver at the peak of the disease, following subcutaneous infection. This may be secondary to the effect of increased levels of ISG expression resulting in protection of the peripheral organs as well as lower levels of virus in the CNS of B6 WT compared to IFNAR KO mice. In other words, it is conceivable that the absence of virus in the periphery of B6 WT mice reflects the preferential homing of the virus to the CNS or the more efficient IFN-mediated protection of virus in other organs. Indeed the levels of mRNA for type I IFNs at the peak of clinical disease are strikingly low and suggest that the virus may interfere with the IFN response as reported for other viruses [42,43]. Additional studies will clarify whether the virus in the B6 WT model exclusively infects the nervous system or whether infections extend to peripheral tissues where it is rapidly cleared and assess whether it extends to the eye as has been reported in the IFNAR KO models.

The B6 WT model also differs from the IFNAR KO model in the type of cellular infiltration and immune response in the CNS at the peak of infection, with IFNAR KO displaying extensive infiltration of neutrophils and macrophage as well as upregulation of inflammatory genes such as *IL-6*, *TNFa* and *IL-1*, whereas B6 WT mice show cellular infiltrates composed primarily of T cells and upregulation of genes associated with Th1 CD4^+^T cells and IFN-driven cytotoxic CD8^+^ T cell responses. Indeed, the differential influx of immune cells evident in the CNS of B6 WT and IFNAR KO mice may underlie the differential clinical outcome. It is possible that the direct damage that Zika causes to the tissue is only one component of its pathogenesis, while the immune response it elicits may also contribute to the pathology. In recent studies we showed that mice infected with Tacaribe virus develop a meningoencephalitis that is driven by the CD4+ and CD8+ T cell response to the virus as mice lacking T cells do not develop disease despite high levels of virus in the CNS[32]. Similar observations were made in mice infected with Sindbis or West Nile virus [44–46]. While it is possible that the influx of T cells into the CNS of B6 WT plays a key role in controlling the virus and aids in the survival of the host, the presence of foci of degenerating neurons suggests that the influx of CD8+ T cells may be driving the observed neuropathology. Understanding the role the different immune cells play in pathogenesis and anti-viral responses will be critical for rational development of therapeutic and preventive approaches.

Type I IFNs play a key role in the earliest responses to viral infections and it is well known that several Flaviviruses interfere with IFN production or activity [47,48], however other factors such as age, neuronal and astrocyte maturation are likely important factors for the establishment of productive infections as observed with other neurotropic RNA viruses including Sindbis, Chikungunya and Tacaribe virus, where neonate but not adult mice develop productive infections [25,26,49]. Similar resistance to challenge was recently reported in studies with ZIKV in IFN deficient mice [28]. In our studies, the virus replicates in neonatal neural tissue in both B6 WT and IFNAR KO mice, but was present in peripheral tissues only in IFNAR KO mice suggesting that: i) interferons play a role in limiting ZIKV spread but the virus can infect other tissues if the innate immune response to the virus is deficient and ii) the virus can infect immature nervous tissue even in immunocompetent animals. Detailed studies of the kinetics of infection and clearance in other organs of B6 WT and IFNAR KO mice are underway to understand whether the virus can establish and/or clear productive infections in other immunoprivileged sites.

These studies establish and characterize the first contemporary ZIKV animal model in immunocompetent neonatal B6 WT mice. The loss of balance and altered gait observed are consistent with the evidence of neurodegeneration and infiltration by cytotoxic CD8^+^ T cells in the cerebellum of B6 WT mice. The presence of infiltrating CD8+ and CD4+ T cells also suggests that the virus could induce an immune response that triggers a neurodestructive inflammatory response in the CNS. The B6 WT model will allow for studies into the immunopathology of the virus in a milieu that does not exclude one of the main immune paths for resistance to virus infection. Given the breadth of knock out and transgenic strains available on a C57BL/6 background, it will facilitate detailed investigations into the pathogenesis of the disease as well as mechanistic studies for possible therapeutics. While the need to infect mice at day 1 of life may limit its utility in assessing the direct protective effect of vaccines, it allows for conducting vaccination studies in pregnant mice followed by challenges in the offspring. Lastly, since this model entails a 13 day prodromal phase, it provides an opportunity for the testing of potential therapeutics and its non-lethal outcome allows for studies assessing the long term effects of the infection, and offers the option of testing conditions that may lead to reactivation of the disease.

## Materials and Methods

### Mice

C57BL/6 (B6) and C57BL/6-IFNAR^-/-^ (IFNAR KO) mice used in this study were bred as homozygous breeding pairs (>20 generations). Mice were housed in sterile microisolator cages under 12-hour day/night cycle and given food and water ad libitum in the specific pathogen-free, AAALAC accredited animal facility of the U.S. Food and Drug Administration’s Division of Veterinary Medicine (Silver Spring, MD).

### Ethics Statement

This study was carried out in strict accordance with the recommendations in the Public Health Service Policy on Humane care and Use of Laboratory Animals. All protocols involving animals were approved by the Animal Care and Use Committee at US-FDA (Protocol Number: #2016–14).

### ZIKV Preparation

Zika virus PRVABC59 (Puerto Rico strain) used in this study is a contemporary strain that was isolated by CDC from the serum of a ZIKV infected patient who travelled to Puerto Rico in 2015. The complete genome sequence is published (Ref. Gene bank accession # KU501215). The virus stocks used for these studies had a titer of 7.2 log_10_ pfu/mL. Virus stocks from CDC were kindly provided by Maria Rios (Food and Drug Administration).

### ZIKV Infections

All newborn mice were born from pathogen-free parents and inoculated with 2000 PFU or 20,000 PFU as indicated by subcutaneous (s.c.) inoculation. IFNAR KO mice were inoculated at 10 days of life (P10) and C57BL/6 mice were inoculated one day after birth (P1). In some experiments IFNAR KO mice were infected on P1 and P3 and C57BL/6 mice were infected on P10. For experiments tracking survival following ZIKV infection, mice were monitored daily for clinical signs of pathology and weighed every other day to minimize handling. Moribund (unable to access nutrition due to severe paresis and/or respiratory distress) animals were euthanized in accordance with the FDA IACUC guidelines.

### Clinical evaluation

Mice were examined daily for signs of infection and weighed on alternate days. Examination included appearance, stance, and motility. Fig 1C includes a description of the changes observed including the evidence of tremors, hyperactivity (increased motor exertion and excitability), stance (increased spread of hind legs while standing or walking), staggered march (evidence of unusual pauses during movement), limb collapse (refers to the momentary collapse of the limb under the weight of the body), seizures (partial loss of voluntary movement with evidence of stiffness and/or tonic contraction of the hind legs), flaccid paralysis (loss of muscle tone with collapse of the lower extremities).

### Virus quantification

For brain homogenates, infected mice were euthanized by CO_2_ asphyxiation and exsanguinated by trans-cardiac perfusion. Brains were removed aseptically, placed in 2 ml of cold RPMI media (ThermoFisher, Carlsbad, CA) and manually disrupted with ice cold Tenbroeck glass grinders (Wheaton, Millville, NJ) until uniform homogenates were obtained. The cellular fractions were pelleted by centrifugation at 400 x g for 15 min. The supernatants were collected and stored at -80°C prior to virus assay. The pelleted (cellular) fraction was used for flow cytometry analysis (see below). In some experiments the homogenates were directly stored at -80°C and centrifuged after thawing. The supernatants were then used for the assay.

Infectious ZIKV levels were measured as TCID_50_/0.5g of tissue on Vero monolayers using an end-point dilution assay as previously described [50,51]. ZIKV RNA levels were measured using quantitative one step reverse transcriptase PCR to amplify ZIKV genome position 1087 to 1163 based on ZIKV MR 766 strain (GenBank accession no. AY632535) Zika virus RNA transcript levels in the samples were quantified by comparing to a standard curve generated using dilutions of an RNA transcript copy of ZIKV sequence. We used 5 micrograms of isolated total RNA for each sample analyzed and ZIKV RNA levels are expressed as ZIKV copies/mL using a standard curve (S4 Fig). The assay can detect all known genotypes of ZIKV and does not cross react with closely related viruses and was performed as described[50]. Briefly, the amplification was performed using 25 ul volume in the Applied Biosystem Viia7 real time PCR machine with the following cycles and conditions: 1^st^ cycle 60C for 30 min followed by 95C for 15 min. The ensuing 45 cycles used 95°C 15 sec and 60°C for 1 min.

### Real-time PCR and Taqman Low Density Arrays (TLDA)

Brains were collected from infected animals that were exsanguinated by transcardiac perfusion with 10 ml ice-cold PBS. The brains were then bisected along the longitudinal fissure and the entire hemisphere of one half brain was flash frozen in liquid N_2_ and stored at -80°C. The frozen tissue was homogenized in 2 mL/half-brain of Trizol reagent (ThermoFisher, Carlsbad, CA) and RNA was isolated following the manufacturers’ protocol and resuspended in molecular grade ultra-pure ddH_2_O. The concentration and purity of isolated RNA was determined by spectrophotometry at 260 nm and 280 nm using a NanoDrop 1000 spectrophotometer (ThermoFisher, Carlsbad, CA). To eliminate potential genomic DNA contamination, the DNA-free Turbo kit (ThermoFisher, Carlsbad, CA) was used as per the manufacturers protocol. Reverse transcription was performed on 1 μg of total RNA, using Multiscript High Capacity Reverse Transcriptase (ThermoFisher, Carlsbad, CA), per the manufacturer’s protocol, using random primers. The resulting cDNA was diluted ten-fold with ultra-pure water and stored at -20°C prior to use in real-time Taqman PCR reactions (ThermoFisher, Carlsbad, CA).

Mouse Immune Array TLDA cards (ThermoFisher, Carlsbad, CA) were used as per manufacturers’ instructions. Briefly, the cDNA generated above was diluted 2-fold. Equal volumes of diluted cDNA and 2x Universal Taqman Master Mix was prepared. This mixture was loaded into the chambers of a Taqman Array card, which was centrifuged to distribute the cDNA throughout the card. IFN and interferon stimulated genes (ISGs) expression levels were measured using individual Taqman gene assays for IFN-β, ISG-15, OAS-1g (ThermoFisher, Carlsbad, CA). The cDNA used in the TLDA reactions was also used for these single gene expression assays, with each sample being run in triplicate for each gene. Fold-change in gene expression was determined using the ΔΔCt method [52], with expression normalized to the expression of the house keeping gene GAPDH. Gene expression is expressed as fold change relative to the indicated uninfected controls. Real-time PCR acquisition and analysis was performed using a Viia7 real-time PCR machine using Quant Studio software, using automatic threshold and endpoint settings (ThermoFisher, Carlsbad, CA).

### Immunofluorescence Immunohistochemistry (IF-IHC) and histology

Brains were removed from exsanguinated Zika infected mice or age-matched uninfected control mice, as described above. The brains were then bisected along the longitudinal fissure and one-half of the brain was submerged in 10% neutral buffered formalin (in phosphate buffered saline (PBS), pH 7.4). The other half was flash frozen in liquid N_2_ for RNA isolation of quantification of virus. After more than 24 hours in fixative, the half-brains were rinsed with PBS and submerged 30% sucrose for cyro-protection. The brains remained in sucrose until they sank in the solution (typically 24–48 hours). Sucrose saturated tissue was then embedded in TissueTek O.C.T (Sakura-Finetek, Torrance, CA) by freezing the brains in Tissue-Tek O.C.T. (Sakura-Finetek, Torrance, CA) embedding compound using 2 methyl butane cooled with dry ice. The brains were wrapped in aluminum foil and stored at -80°C. Sagittal sections (20 μm thick) were cut using a Leica CM1900 cryostat (Leica Biosystems, Buffalo Grove, IL) and thaw-mounted onto SuperFrost-plus microscope slides (Fisher Scientific, Carlsbad, CA). The sections were stored at -80°C until staining. Prior to staining, sections were thawed and dried at room temperature (RT) for approximately 10 min, rinsed with phosphate buffered saline (PBS) and permeabilized using 0.2% Tween-20 in PBS for 20 minutes at RT. For ZIKV staining, antigen unmasking was performed prior to staining by submerging the sections in Sodium citrate solution (pH8.8, 80°C) for 30 minute. These sections were then blocked with 1% low fat milk in PBS + 0.05% Tween-20 for 1 hour prior to staining. All other sections were blocked with 5% normal goat serum + 1% bovine serum albumin (BSA) in PBS with 0.05% Tween-20 for at least 60 minutes at RT. Primary antibodies used include: mouse anti-flavivirus mAb (cloneD1- 4G2-4-15; EMD Millipore), human polyclonal anti-zika virus envelope antibody (Kerafast EVU302, MA), neurofilament heavy chain cocktail [SMI31 and SMI32 mAb] (Biolegend) and rat anti-CD45 (BD Biosciences). Tissue sections were incubated overnight in a humidified chamber at 4°C with primary antibodies diluted with 1% BSA in PBS with 0.05% Tween-20. The slides were then rinsed with PBS and incubated with the appropriate AlexaFluor-conjugated (raised in goat) secondary antibodies, diluted in 1% BSA in PBS with 0.05% Tween-20 (ThermoFisher, Carlsbad, CA) for > 60 min at RT. All IF-IHC sections were mounted with ProLong Diamond anti-fade mounting media containing DAPI (ThermoFisher, Carlsbad, CA).Fluorescently labelled antibodies were detected at emission wavelengths: 405 (DAPI), 535 (Alexa-fluor 488, Fluoro-Jade C), 605 (Alexa-fluor 568). Sections were imaged using a Pannoramic Digital Slide Scanner. Images were captured using Pannoramic Viewer software (3DHistech, Budapest, HUN). Fluoro-Jade C histology staining, which specifically detects neuronal degeneration,[53] was performed as per the manufacturers protocol (EMD Millipore, Billerica, MA). For confocal imaging, sections were imaged using a Zeiss LSM 880 confocal microscope, using a 405 nm, 488 nm, and 561 nm excitation lasers. Optimal fluorescence detection settings were determined using B6 WT sections and applied to all other sections. Images were acquired using the Zeiss Zen software using a Z-stack, with slices of 0.44–0.49 microns per slice for all sections. Laser and PMT setting were consistent for uninfected and infected sections. Maximum intensity projections of these Z-stacks were generated using the Zen software. All images were prepared for publication using Adobe Photoshop CC 2015 software.

### Flow Cytometry

The cellular fractions of brain homogenates (see Virus Quantification) were pooled and resuspended in 30% percoll (GE Life Sciences, Marlborough, MA) in RPMI + 25 mM HEPES (ThermoFisher, Carlsbad, CA) and underlayed with 1 ml of 70% percoll. After centrifugation at 800 x g for 30 minutes, CNS cells were collected from the 30%-70% interface, washed in RPMI and isolated by centrifugation at 400 x g for 10 minutes.

Non-specific antibody binding was blocked with a mix of mouse F_c_ block (purified α-CD16/32, BD Biosciences) and normal mouse serum for at least 15 minutes. All antibodies (anti-CD45, anti-CD4, anti-CD8, anti-CD11b, anti-CD19 anti-Ly6G, anti-NK1.1 (BD Biosciences, San Jose, CA)); and anti-F4/80 (AbD Serotec, Raleigh, NC) were directly conjugated with one of the following fluorochromes: fluorescein isothiocyanate (FITC), phycoerythrin (PE), peridinin-chlorophyll proteins (PerCP), or alophycocyanin (APC). The cells were incubated with these antibodies for 20 min in FACS buffer (1% BSA in PBS), then washed with FACS buffer, fixed in 2% paraformaldehyde solution and acquired using a BD Fortessa flow cytometer (BD Biosciences, San Jose, CA). The flow cytometer was calibrated using beads (eBiosciences) conjugated with fluorescent antibodies for each channel in the assay. The resulting data from each samples was analysed using FlowJo (version 10) software (FlowJo, LLC, Ashland, OR).

## Supporting Information

S1 Movie Clinical presentation of non-fatal encephalitis in B6 WT mice following ZIKV infection. B6 WT mice were infected subcutaneously with 2x10^3^TCID50 ZIKV at P1. They remained asymptomatic until P13. **S1 Movie** shows P13-15 B6 WT mouse taken with a mobile phone. No editing of the videos was performed. All mice in the cohort show a similar phenotype. Note the stance of the animal and loss of balance.
(MOV)

S2 Movie Clinical presentation of non-fatal encephalitis in B6 WT mice following ZIKV infection. B6 WT mice were infected subcutaneously with 2x10^3^TCID50 ZIKV at P1. They remained asymptomatic until P13. S2 Movie shows P13-15 B6 WT mice taken with a mobile phone. No editing of the videos was performed. All mice in the cohort show a similar phenotype. Note the asymmetric tonic extension of the hind leg and the difficulty in turning to the upright position.
(MOV)

S3 Movie Clinical presentation of non-fatal encephalitis in B6 WT mice following ZIKV infection. B6 WT mice were infected subcutaneously with 2x10^3^TCID50 ZIKV at P1. They remained asymptomatic until P13. S3 Movie shows P13-15 B6 WT mice taken with a mobile phone. No editing of the videos was performed. All mice in the cohort show a similar phenotype. Note the stance of the animal with weakened limbs and loss of balance as well as the claudication of the limbs.
(MOV)

S4 Movie Clinical presentation of fatal encephalitis in IFNAR KO mice following ZIKV infection. IFNAR KO mice were infected subcutaneously with 2x10^3^TCID50 ZIKV at P10. The mice remained asymptomatic until P14 when they develop bilateral flaccid paralysis of the hind legs. The animals died 24 hours after developing paralysis.
(MOV)

S1 Fig Infection of IFNAR KO mice on days 1, 3, and 10 of life with ZIKV PRV ABC59 (2x10^3^pfu sc). Note that the mice succumb 5 days post infection regardless of the challenge day. Mice showed paralysis of the hind limbs 6–12 hours prior to death.
(TIF)

S2 Fig ZIKV infection of neurons in hippocampus region. Maximum projection of confocal micrograph from ZIKV infected, B6 WT mouse. Sections were stained with anti-ZIKV pAb (green) and anti-neurofilament heavy chain (NF, red). Overlay of these two stains indicates infected neurons (yellow). Image isolated from within the hippocampus, proximal to the lateral ventricle (LV). Rostral (R)-caudal (C) orientation of the brain indicated. Scale bar = 50 μm.
(TIF)

S3 Fig Expression of apoptosis related genes in CNS of ZIKV infected mice. Mouse inflammation TLDA (Applied Biosystems) analysis comparing ZIKV infected B6 WT (red) and IFNAR KO (blue) in CNS at P16 (15 dpi and 5 dpi, respectively). The table shows the geometric mean and SEM of the fold increase in expression of genes related to apoptosis. Note that none of the genes shows an upregulation larger then 10 fold over uninfected animals and no significant difference was evident between in B6 WT and IFNAR KO mice.
(PDF)

S4 Fig Standard curve used to calculate the viral copy number. The standard curve was generated using dilutions of an RNA transcript copy of ZIKV sequence. The amplification was performed using 25 ul volume in the Applied Biosystem Viia7 real time PCR machine with the following cycles and conditions: The 1^st^ cycle 60C for 30 min followed by 95C for 15 min. The ensuing 45 cycles used 95C 15 sec and 60 for 1 min.
(TIFF)

## References

[1] Dick GWA (1952) Zika virus (II). Pathogenicity and physical properties. Transactions of the Royal Society of Tropical Medicine and Hygiene 46: 521–534. 12995441 10.1016/0035-9203(52)90043-6

[2] Hofer U (2016) Viral Pathogenesis: Tracing the steps of Zika virus. Nat Rev Micro 14: 401–401. 10.1038/nrmicro.2016.80 27211788

[3] WHO (2016) One year into the Zika outbreak: how an obscure disease beame a global health emergency: 2. The first outbreaks. http://www.who.int/emergencies/zika-virus/articles/one-year-outbreak/en/index1.html: World Health Organization.

[4] Cao-Lormeau V-M, Blake A, Mons S, Lastère S, Roche C, et al (2016) Guillain-Barré Syndrome outbreak associated with Zika virus infection in French Polynesia: a case-control study. The Lancet 387: 1531–1539. 10.1016/S0140-6736(16)00562-6 PMC5444521 26948433

[5] Bautista LE, Sethi AK Association between Guillain-Barré syndrome and Zika virus infection. The Lancet 387: 2599–2600. 10.1016/S0140-6736(16)30844-3 27353815

[6] Sejvar JJ, Baughman AL, Wise M, Morgan OW (2011) Population incidence of Guillain-Barre syndrome: a systematic review and meta-analysis. Neuroepidemiology 36: 123–133. 10.1159/000324710 21422765 PMC5703046

[7] WHO (2016) Zika Situation Report, February 26, 2016. http://who.int.ezproxy.nihlibrary.nih.gov/emergencies/zika-virus/situation-report/26-february-2016/en/: World Heath Organization.

[8] WHO (2016) WHO Director-General summarizes the outcome of the emergency committee regarding clusters of microencephaly and Gullain Barre Syndrome. http://www.who.int.ezproxy.nihlibrary.nih.gov/mediacentre/news/statements/2016/emergency-committee-zika-microcephaly/en/: World Health Organization.

[9] Paploski IA, Prates AP, Cardoso CW, Kikuti M, Silva MM, et al (2016) Time Lags between Exanthematous Illness Attributed to Zika Virus, Guillain-Barre Syndrome, and Microcephaly, Salvador, Brazil. Emerg Infect Dis 22. 10.3201/eid2208.160496 PMC4982160 27144515

[10] Garcez PP, Loiola EC, Madeiro da Costa R, Higa LM, Trindade P, et al (2016) Zika virus impairs growth in human neurospheres and brain organoids. Science 352: 816–818. 10.1126/science.aaf6116 27064148

[11] Qian X, Nguyen HN, Song MM, Hadiono C, Ogden SC, et al (2016) Brain-Region-Specific Organoids Using Mini-bioreactors for Modeling ZIKV Exposure. Cell 165: 1238–1254. 10.1016/j.cell.2016.04.032 27118425 PMC4900885

[12] Rasmussen SA, Jamieson DJ, Honein MA, Petersen LR (2016) Zika Virus and Birth Defects—Reviewing the Evidence for Causality. New England Journal of Medicine 374: 1981–1987. 10.1056/NEJMsr1604338 27074377

[13] Wu K-Y, Zuo G-L, Li X-F, Ye Q, Deng Y-Q, et al (2016) Vertical transmission of Zika virus targeting the radial glial cells affects cortex development of offspring mice. Cell Res 26: 645–654. 10.1038/cr.2016.58 27174054 PMC4897185

[14] Lazear Helen M, Govero J, Smith Amber M, Platt Derek J, Fernandez E, et al A Mouse Model of Zika Virus Pathogenesis. Cell Host & Microbe. 10.1016/j.chom.2016.03.010 PMC4866885 27066744

[15] Aliota MT, Caine EA, Walker EC, Larkin KE, Camacho E, et al (2016) Characterization of Lethal Zika Virus Infection in AG129 Mice. PLoS Negl Trop Dis 10: e0004682 10.1371/journal.pntd.0004682 27093158 PMC4836712

[16] Winkelmann ER, Luo H, Wang T (2016) West Nile Virus Infection in the Central Nervous System. F1000Res 5. 10.12688/f1000research.7404.1 PMC4755400 26918172

[17] Cugola FR, Fernandes IR, Russo FB, Freitas BC, Dias JL, et al (2016) The Brazilian Zika virus strain causes birth defects in experimental models. Nature 534: 267–271. 10.1038/nature18296 27279226 PMC4902174

[18] Li H, Saucedo-Cuevas L, Regla-Nava Jose A, Chai G, Sheets N, et al (2016) Zika Virus Infects Neural Progenitors in the Adult Mouse Brain and Alters Proliferation. Cell Stem Cell. 10.1016/j.stem.2016.08.005 PMC5097023 27545505

[19] Miner JJ, Cao B, Govero J, Smith AM, Fernandez E, et al (2016) Zika Virus Infection during Pregnancy in Mice Causes Placental Damage and Fetal Demise. Cell 165: 1081–1091. 10.1016/j.cell.2016.05.008 27180225 PMC4874881

[20] Miner Jonathan J, Sene A, Richner Justin M, Smith Amber M, Santeford A, et al (2016) Zika Virus Infection in Mice Causes Panuveitis with Shedding of Virus in Tears. Cell Reports. 10.1016/j.celrep.2016.08.079 PMC5040391 27612415

[21] Mysorekar IU, Diamond MS (2016) Modeling Zika Virus Infection in Pregnancy. New England Journal of Medicine 375: 481–484. 10.1056/NEJMcibr1605445 27433842

[22] Semple BD, Blomgren K, Gimlin K, Ferriero DM, Noble-Haeusslein LJ (2013) Brain development in rodents and humans: Identifying benchmarks of maturation and vulnerability to injury across species. Progress in neurobiology 0: 1–16. 10.1016/j.pneurobio.2013.04.001 PMC3737272 23583307

[23] Pletnikov MV, Rubin SA, Moran TH, Carbone KM (2003) Exploring the cerebellum with a new tool: neonatal Borna disease virus (BDV) infection of the rat's brain. Cerebellum 2: 62–70. 10.1080/14734220309425 12882236

[24] Amstey MS, Kobos K (1976) An experimental model for disseminated herpesvirus infection of the neonate. Am J Obstet Gynecol 125: 40–44. 179321 10.1016/0002-9378(76)90888-7

[25] Pedras-Vasconcelos JA, Puig M, Sauder C, Wolbert C, Ovanesov M, et al (2008) Immunotherapy with CpG Oligonucleotides and Antibodies to TNF- Rescues Neonatal Mice from Lethal Arenavirus-Induced Meningoencephalitis. The Journal of Immunology 180: 8231–8240. 18523289 10.4049/jimmunol.180.12.8231

[26] Couderc T, Chretien F, Schilte C, Disson O, Brigitte M, et al (2008) A mouse model for Chikungunya: young age and inefficient type-I interferon signaling are risk factors for severe disease. PLoS Pathog 4: e29 10.1371/journal.ppat.0040029 18282093 PMC2242832

[27] Dowall SD, Graham VA, Rayner E, Atkinson B, Hall G, et al (2016) A Susceptible Mouse Model for Zika Virus Infection. PLoS Negl Trop Dis 10: e0004658 10.1371/journal.pntd.0004658 27149521 PMC4858159

[28] Lazear HM, Govero J, Smith AM, Platt DJ, Fernandez E, et al (2016) A Mouse Model of Zika Virus Pathogenesis. Cell Host Microbe 19: 720–730. 10.1016/j.chom.2016.03.010 27066744 PMC4866885

[29] Rossi SL, Tesh RB, Azar SR, Muruato AE, Hanley KA, et al (2016) Characterization of a Novel Murine Model to Study Zika Virus. Am J Trop Med Hyg 94: 1362–1369. 10.4269/ajtmh.16-0111 27022155 PMC4889758

[30] Bayer A, Lennemann Nicholas J, Ouyang Y, Bramley John C, Morosky S, et al (2016) Type III Interferons Produced by Human Placental Trophoblasts Confer Protection against Zika Virus Infection. Cell Host & Microbe 19: 705–712. 27066743 10.1016/j.chom.2016.03.008 PMC4866896

[31] Miner Jonathan J, Cao B, Govero J, Smith Amber M, Fernandez E, et al (2016) Zika Virus Infection during Pregnancy in Mice Causes Placental Damage and Fetal Demise. Cell 165: 1081–1091. 10.1016/j.cell.2016.05.008 27180225 PMC4874881

[32] Ireland DDC, Tami C, Pedras-Vasconcelos J, Verthelyi D (2016) CD4 and CD8 T cells mediate distinct lethal meningoencephalitis in mice challenged with Tacaribe arenavirus. Cell Mol Immunol. 10.1038/cmi.2016.41 PMC5214944 27569560

[33] Ida-Hosonuma M, Iwasaki T, Yoshikawa T, Nagata N, Sato Y, et al (2005) The Alpha/Beta Interferon Response Controls Tissue Tropism and Pathogenicity of Poliovirus. Journal of Virology 79: 4460–4469. 10.1128/JVI.79.7.4460-4469.2005 15767446 PMC1061561

[34] Zhao L, Rose KM, Elliott R, Van Rooijen N, Weiss SR (2011) Cell-Type-Specific Type I Interferon Antagonism Influences Organ Tropism of Murine Coronavirus. Journal of Virology 85: 10058–10068. 10.1128/JVI.05075-11 21752905 PMC3196400

[35] Bell TM, Field EJ, Narang HK (1971) Zika virus infection of the central nervous system of mice. Arch Gesamte Virusforsch 35: 183–193. 5002906 10.1007/BF01249709

[36] Way JH, Bowen ET, Platt GS (1976) Comparative studies of some African arboviruses in cell culture and in mice. J Gen Virol 30: 123–130. 10.1099/0022-1317-30-1-123 1245842

[37] Nazmi A, Dutta K, Das S, Basu A (2011) Japanese encephalitis virus-infected macrophages induce neuronal death. J Neuroimmune Pharmacol 6: 420–433. 10.1007/s11481-011-9271-x 21424747

[38] Frumence E, Roche M, Krejbich-Trotot P, El-Kalamouni C, Nativel B, et al (2016) The South Pacific epidemic strain of Zika virus replicates efficiently in human epithelial A549 cells leading to IFN-β production and apoptosis induction. Virology 493: 217–226. 10.1016/j.virol.2016.03.006 27060565

[39] Herz J, Johnson KR, McGavern DB (2015) Therapeutic antiviral T cells noncytopathically clear persistently infected microglia after conversion into antigen-presenting cells. J Exp Med 212: 1153–1169. 10.1084/jem.20142047 26122661 PMC4516789

[40] Hamel R, Dejarnac O, Wichit S, Ekchariyawat P, Neyret A, et al (2015) Biology of Zika Virus Infection in Human Skin Cells. J Virol 89: 8880–8896. 10.1128/JVI.00354-15 26085147 PMC4524089

[41] Jin R, Zhu W, Cao S, Chen R, Jin H, et al (2013) Japanese encephalitis virus activates autophagy as a viral immune evasion strategy. PLoS One 8: e52909 10.1371/journal.pone.0052909 23320079 PMC3540057

[42] Chang T-H, Kubota T, Matsuoka M, Jones S, Bradfute SB, et al (2009) Ebola Zaire Virus Blocks Type I Interferon Production by Exploiting the Host SUMO Modification Machinery. PLoS Pathog 5: e1000493 10.1371/journal.ppat.1000493 19557165 PMC2696038

[43] Borrow P, Martínez-Sobrido L, de la Torre JC (2010) Inhibition of the Type I Interferon Antiviral Response During Arenavirus Infection. Viruses 2: 2443–2480. 10.3390/v2112443 21994626 PMC3185579

[44] Wang Y, Lobigs M, Lee E, Mullbacher A (2003) CD8+ T cells mediate recovery and immunopathology in West Nile virus encephalitis. J Virol 77: 13323–13334. 10.1128/JVI.77.24.13323-13334.2003 14645588 PMC296062

[45] Kulcsar KA, Griffin DE (2016) T cell-derived interleukin-10 is an important regulator of the Th17 response during lethal alphavirus encephalomyelitis. J Neuroimmunol 295–296: 60–67. 10.1016/j.jneuroim.2016.04.010 27235350 PMC4884611

[46] Kulcsar KA, Baxter VK, Greene IP, Griffin DE (2014) Interleukin 10 modulation of pathogenic Th17 cells during fatal alphavirus encephalomyelitis. Proc Natl Acad Sci U S A 111: 16053–16058. 10.1073/pnas.1418966111 25362048 PMC4234572

[47] Lin RJ, Chang BL, Yu HP, Liao CL, Lin YL (2006) Blocking of interferon-induced Jak-Stat signaling by Japanese encephalitis virus NS5 through a protein tyrosine phosphatase-mediated mechanism. J Virol 80: 5908–5918. 10.1128/JVI.02714-05 16731929 PMC1472572

[48] Green AM, Beatty PR, Hadjilaou A, Harris E (2014) Innate Immunity to Dengue Virus Infection and Subversion of Antiviral Responses. Journal of Molecular Biology 426: 1148–1160. 10.1016/j.jmb.2013.11.023 24316047 PMC4174300

[49] Lustig S, Jackson AC, Hahn CS, Griffin DE, Strauss EG, et al (1988) Molecular basis of Sindbis virus neurovirulence in mice. J Virol 62: 2329–2336. 2836615 10.1128/jvi.62.7.2329-2336.1988 PMC253389

[50] Lanciotti RS, Kosoy OL, Laven JJ, Velez JO, Lambert AJ, et al (2008) Genetic and serologic properties of Zika virus associated with an epidemic, Yap State, Micronesia, 2007. Emerg Infect Dis 14: 1232–1239. 10.3201/eid1408.080287 18680646 PMC2600394

[51] Pedras-Vasconcelos JA, Goucher D, Puig M, Tonelli LH, Wang V, et al (2006) CpG oligodeoxynucleotides protect newborn mice from a lethal challenge with the neurotropic Tacaribe arenavirus. J Immunol 176: 4940–4949. 16585590 10.4049/jimmunol.176.8.4940

[52] Livak KJ, Schmittgen TD (2001) Analysis of Relative Gene Expression Data Using Real-Time Quantitative PCR and the 2-[Delta][Delta]CT Method. Methods 25: 402–408. 10.1006/meth.2001.1262 11846609

[53] Schmued LC, Hopkins KJ (2000) Fluoro-Jade B: a high affinity fluorescent marker for the localization of neuronal degeneration. Brain Res 874: 123–130. 10960596 10.1016/s0006-8993(00)02513-0

