# Supplementary figures and images for: Zika (PRVABC59) Infection Is Associated with T cell Infiltration and Neurodegeneration in CNS of Immunocompetent Neonatal C57Bl/6 Mice
Source: PLoS Pathog. 2016 Nov 17;12(11):e1006004. doi: 10.1371/journal.ppat.1006004 (PMC5113993; doi:10.1371/journal.ppat.1006004)

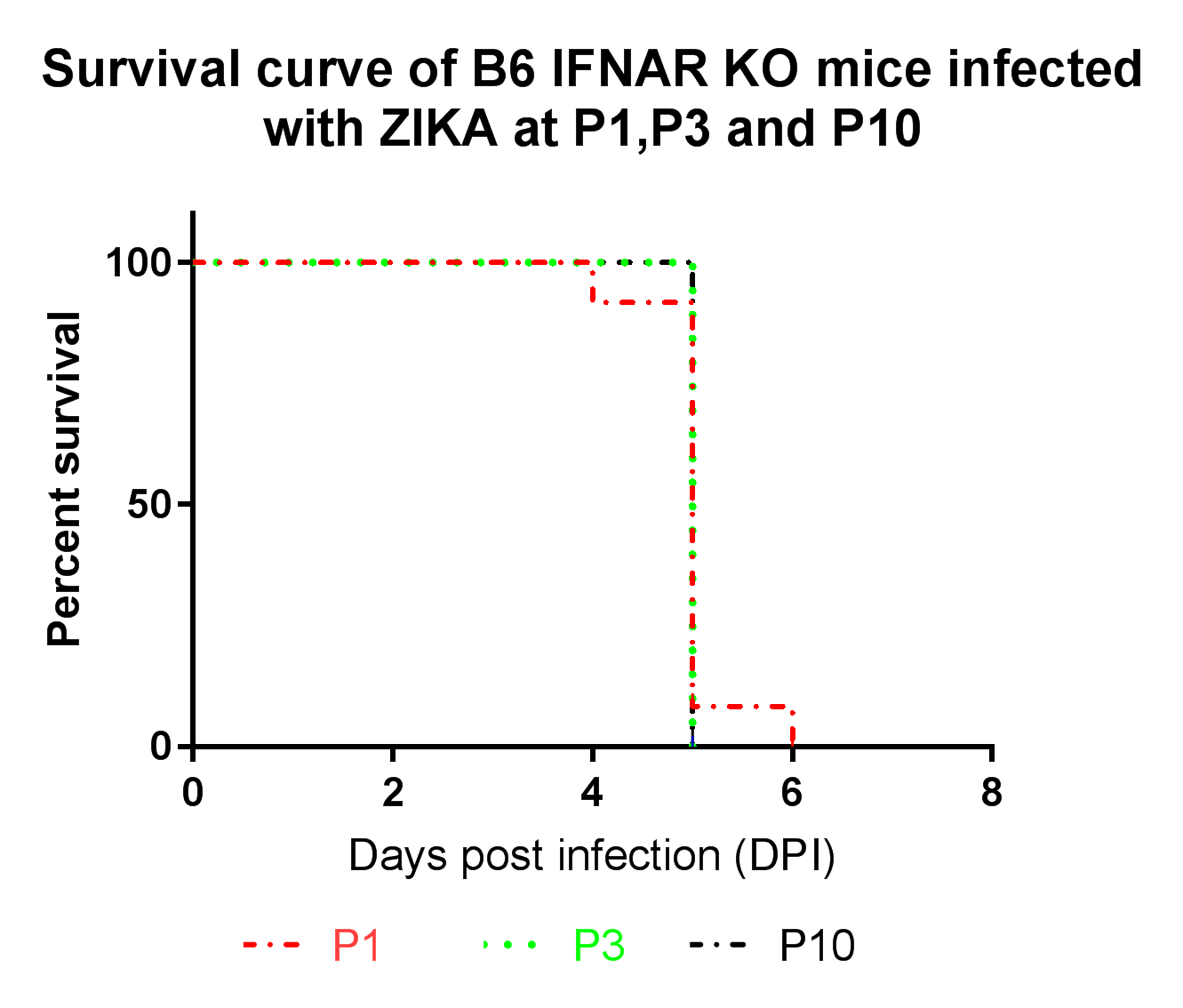

Supplement: S1 Fig — Note that the mice succumb 5 days post infection regardless of the challenge day. Mice showed paralysis of the hind limbs 6–12 hours prior to death. (TIF) [file ppat.1006004.s005.tif]

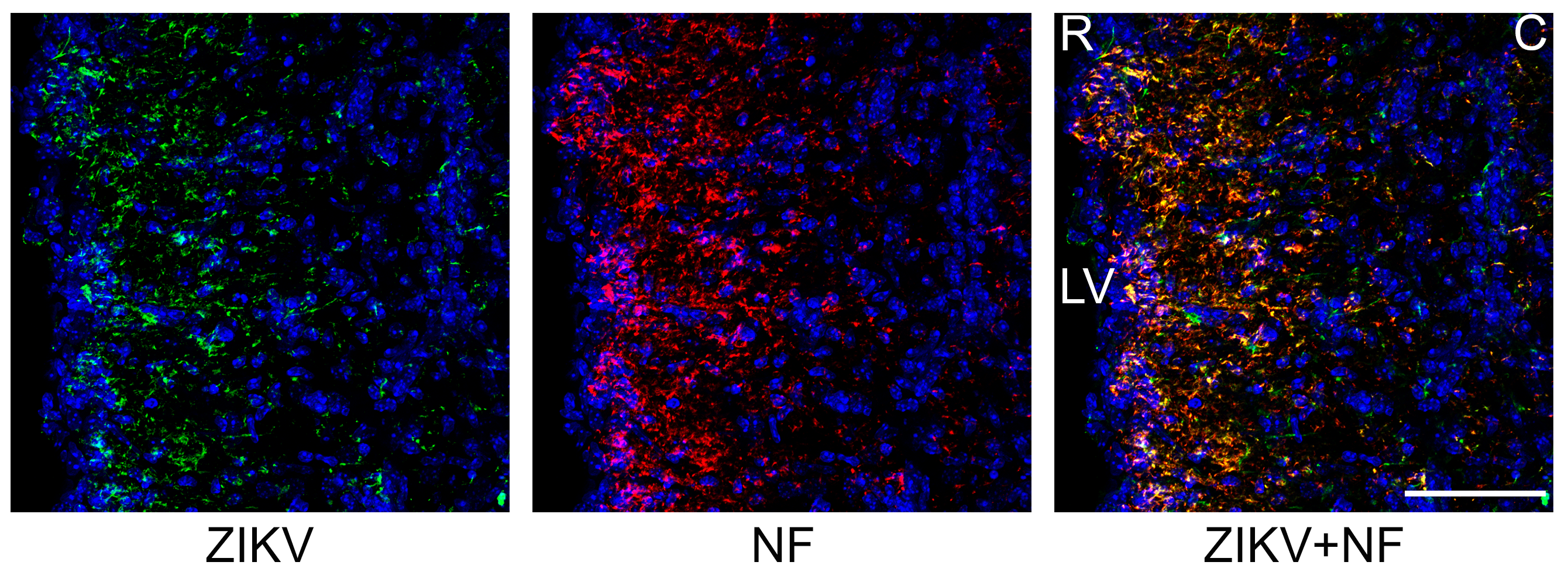

Supplement: S2 Fig — Maximum projection of confocal micrograph from ZIKV infected, B6 WT mouse. Sections were stained with anti-ZIKV pAb (green) and anti-neurofilament heavy chain (NF, red). Overlay of these two stains indicates infected neurons (yellow). Image isolated from within the hippocampus, proximal to the lateral ventricle (LV). Rostral (R)-caudal (C) orientation of the brain indicated. Scale bar = 50 μm. (TIF) [file ppat.1006004.s006.tif]

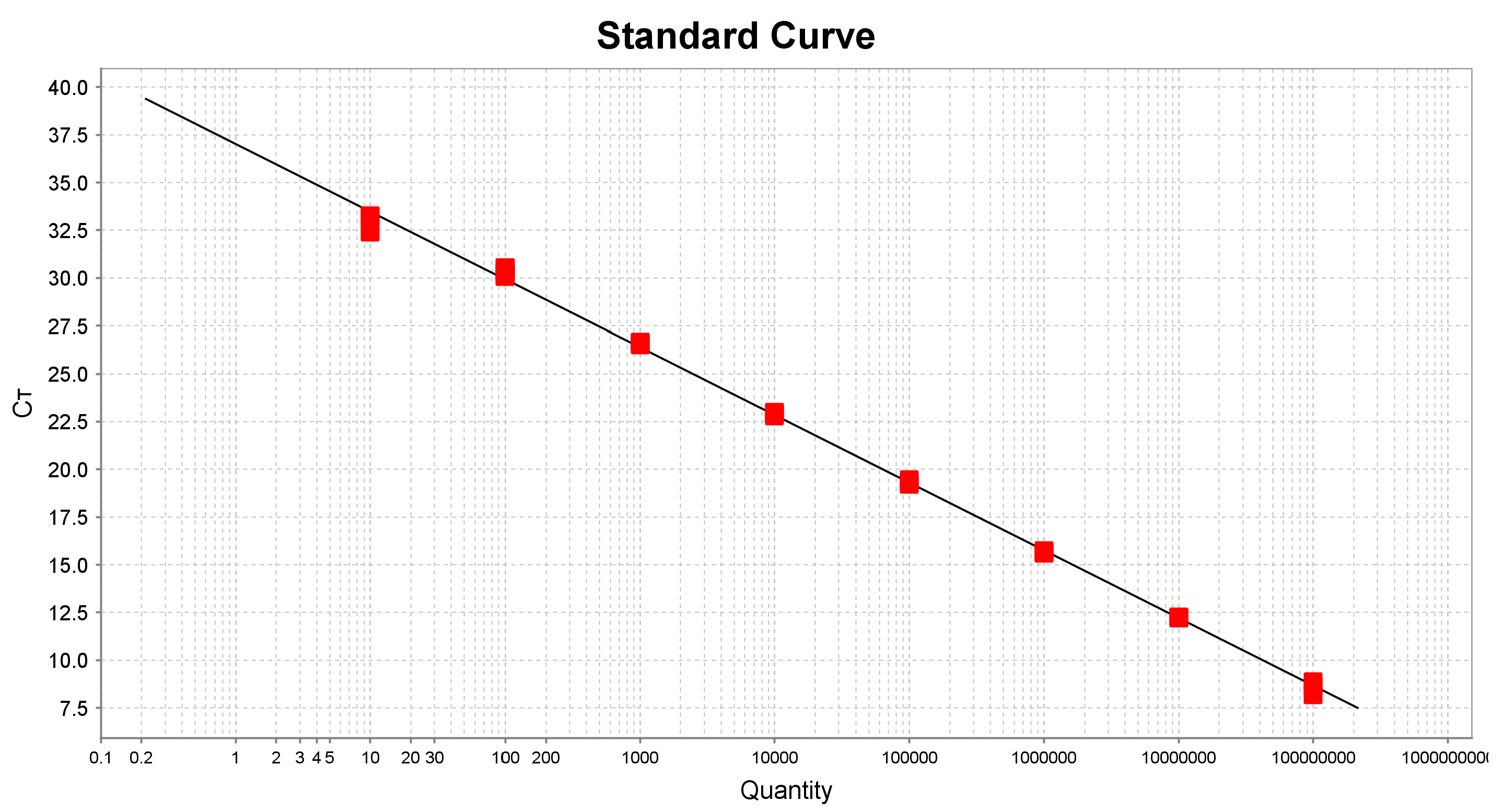

Supplement: S4 Fig — The standard curve was generated using dilutions of an RNA transcript copy of ZIKV sequence. The amplification was performed using 25 ul volume in the Applied Biosystem Viia7 real time PCR machine with the following cycles and conditions: The 1st cycle 60C for 30 min followed by 95C for 15 min. The ensuing 45 cycles used 95C 15 sec and 60 for 1 min. (TIFF) [file ppat.1006004.s008.tiff]
