# Supplementary material for: Zika (PRVABC59) Infection Is Associated with T cell Infiltration and Neurodegeneration in CNS of Immunocompetent Neonatal C57Bl/6 Mice
Source: PLoS Pathog. 2016 Nov 17;12(11):e1006004. doi: 10.1371/journal.ppat.1006004 (PMC5113993; doi:10.1371/journal.ppat.1006004)
Supplement: S3 Fig — Mouse inflammation TLDA (Applied Biosystems) analysis comparing ZIKV infected B6 WT (red) and IFNAR KO (blue) in CNS at P16 (15 dpi and 5 dpi, respectively). The table shows the geometric mean and SEM of the fold increase in expression of genes related to apoptosis. Note that none of the genes shows an upregulation larger then 10 fold over uninfected animals and no significant difference was evident between in B6 WT and IFNAR KO mice. (PDF) [file ppat.1006004.s007.pdf]

	B6 WT	SEM	IFNAR KO	SEM
Agtr2	1.0	0.1	1.5	0.2
Bax	1.3	0.1	1.4	0.2
Bcl2	1.4	0.1	1.1	0.1
Bcl2l1	1.1	0.1	1.3	0.1
Fas	2.0	0.4	6.1	0.6
Fasl	10.2	2.5	8.4	2.7
